# Comparison of different diagnostic techniques for the detection of cryptosporidiosis in bovines

**DOI:** 10.14202/vetworld.2016.211-215

**Published:** 2016-02-27

**Authors:** K. M. H. Rekha, G. C. Puttalakshmamma, Placid E. D’Souza

**Affiliations:** 1 Department of Animal Husbandary & Veterinary Services, Government of Karnataka, Muthur, Piriapatna, Mysore, Karnataka, India; 2 Department of Parasitology, Veterinary College, Hebbal, Bengaluru, Karnataka, India; 3 Department of Veterinary Parasitology, Veterinary College, Hassan, Karnataka, India

**Keywords:** bovines, *Cryptosporidium*, Kinyoun's acid-fast method, modified Ziehl–Neelsen, oocysts, safranin-methylene blue staining, SSF, diagnostic methods

## Abstract

**Aim:**

Aim of the present study was to compare different methods, *viz*., Sheather's sugar flotation (SSF), Ziehl-Neelsen (ZN), Kinyoun's acid-fast method (KAF), safranin-methylene blue staining (SMB), and negative staining techniques such as nigrosin staining, light green staining, and malachite green staining for the detection of *Cryptosporidium* spp. oocysts in bovines.

**Materials and Methods:**

A total of 455 fecal samples from bovines were collected from private, government farms and from the clinical cases presented to Department of Medicine, Veterinary College, Bengaluru. They were subjected for SSF, ZN, KAF, SMB and negative staining methods.

**Results:**

Out of 455 animal fecal samples screened 5.71% were found positive for *Cryptosporidium* spp. oocysts. The species were identified as *Cryptosporidium parvum* in calves and *Cryptosporidium andersoni* in adults based on the morphological characterization and micrometry of the oocysts.

**Conclusions:**

Of all the techniques, fecal flotation with sheather's was found to be more specific and sensitive method for the detection of *Cryptosporidium* spp. oocysts. Among the conventional staining methods, the SMB gives better differentiation between oocysts and yeast. Among the three negative staining methods, malachite green was found sensitive over the other methods.

## Introduction

Cryptosporidiosis is a protozoan disease caused by obligate intestinal coccidian parasite belonging to the genus *Cryptosporidium* that infects more than 150 hosts including human beings. In India, *Cryptosporidium* *andersoni* in cattle was the first reported in 2004 [1], whereas *Cryptosporidium ryanae* was recently reported in calves by Venu from Tamil Nadu [2]. Eventhough, *C. andersoni* does not lead to any clinical disease in cattle, the shedding of oocysts in the feces is responsible for environmental contamination, thus acts as a source of infection. Cryptosporidiosis in calves is characterized by acute gastrointestinal disturbances, mucoid or hemorrhagic watery diarrhea, fever, lethargy, anorexia, and weight loss leading to significant economic losses to the livestock sector [3].

A diagnosis of cryptosporidiosis is based on the identification of *Cryptosporidium* spp. oocysts in the fecal sample by conventional and immunodiagnostic methods. Several conventional techniques such as flotation by Sheather's sugar/zinc sulfate solution, formal ether concentration method, formal ethyl acetate sedimentation technique, Ziehl–Neelsen (ZN), Kinyoun's acid-fast method (KAF) and some of negative staining methods using Nigrosin, light green, malachite green and carbol fuchsin are used for the diagnosis of cryptosporidiosis. Molecular techniques like polymerase chain reaction (PCR) are widely used nowadays for the genotyping of cryptosporidiosis.

The present study was undertaken to compare the different techniques for the identification of *Cryptosporidium* spp. oocysts in the fecal sample of bovines.

## Materials and Methods

### Ethical approval

Freshly passed faecal samples were collected immediately after voiding without any stress or harm to the animals.

### Collection of faecal samples

The 455 fecal samples from bovines were collected from five organized dairy farms and Veterinary hospitals located in and around Bengaluru were screened during a period of one-year from July 2006 to June 2007. Each sample was studied macroscopically to establish its consistency as liquid, soft or solid and the presence of mucus or blood was noted. Samples were stored at 4°C in a refrigerator until analysis. Later, the samples were preserved in 10% formalin. The fecal samples coated with mucus were treated with 10% KOH [4] for efficient recovery of oocysts.

### Sheather's sugar flotation (SSF) method

The sucrose floatation was carried out by following standard procedure with Sheather's sugar solution of 1.26 specific gravity; centrifugation was done at 500 ×*g* for 10 min. *Cryptosporidium* oocysts were observed under magnification (×40) and oil immersion (×100) for observation of internal morphology.

### ZN staining

A hot and cold method of ZN staining of fecal smears was done as per the procedure [5] with slight modifications. Thin smears of fecal sediment were made on a clean grease free glass slide and air-dried. Then, the smears were fixed transiently over a flame. The smears were then stained with strong carbol fuchsin solution for 5 min. In the hot method, the slide was heated until the steam appeared but boiling was avoided. In cold method, slides were not heated. After staining, the smears were washed in running water for 1-2 min. Then, the slides were subsequently decolorized in 5% sulphuric acid for 30 s and counterstained with 3% methylene blue for 1 min.

### Kinyoun's staining method

Kinyoun's staining was done as per the procedure [6]. The thin smear of fecal sediment was made and air-dried. The smear was fixed with absolute methanol for 1 min. Then, the slide was flooded with Kinyoun's carbol fuchsin stain for 5 min. After staining, the slide was rinsed with 50% ethanol for 3-5 s and later with distilled water. Stained smear decolorized with 1% sulphuric acid for 2 min and was counterstained with 1% methylene blue for 1 min.

### Safranin methylene blue staining (SMB)

In the present study, the staining technique was followed as per method [7]. Thin smears from the fecal sediment were made on the glass slide using a stick. Then, the smears were air-dried and fixed transiently over a flame. Again the smears were fixed in acid alcohol for 3-5 min. Then, the smears were flooded with 1% safranin solution for 60 s along with gentle heating until steam comes. The smears were then washed with water and counterstained with 1% methylene blue for 30 s. Then, the smears were again washed with water air-dried and examined.

### Negative staining method

#### Nigrosin staining

Nigrosin staining technique was done as per the methodology [8] with slight modifications. A drop of fecal sediment and a drop of 1% nigrosin stain were mixed on a glass slide, evenly spread and air-dried.

#### Light green staining

Light green staining technique was done as per the methodology [9] with slight modifications. A drop of fecal sediment and a drop of 1% light green stain were mixed on a glass slide, evenly spread and air-dried.

#### Malachite green staining

Malachite green staining technique was done as per the methodology [10] with slight modifications. A drop of fecal sediment and a drop of 5% malachite green stain were mixed on a glass slide, evenly spread and air-dried. In all the above methods, the smears examined under oil immersion (×100).

### Micrometry identification

Identification of oocyst was done by using bright field microscopy morphological characterization and micrometry [11]. The intensity of infection was estimated based on the oocyst counts on ZN stained smear by counting 20 randomly selected fields at ×100 magnification and average of the oocyst counts were grouped as few, moderate and many as per the procedure [12].

### Statistical analysis

The data were analyzed using Chi-square test and t-test, ANOVA as per the Quilenz *et al*. [13].

## Results and Discussion

Out of 455 fecal samples screened, 26 (5.71%) animals were found positive for the cryptosporidiosis. The prevalence of cryptosporidiosis was a higher in diarrheic animals (24.20%) when compared to non-diarrheic (16.60%), which was statistically significant (p<0.05). In India, the disease was reported in Uttar Pradesh [14] later in Calcutta [15,16], Pondicherry [1] and Andhra Pradesh [17]. During the study, two types of oocysts were found. Micrometrically larger oocysts had a length of 7.2±0.835 μm and width of 5.7±0.835 μm and confirmed as *C. andersoni*, whereas smaller oocysts with average length of 5.2 ±0.422 μm and width of 4.05±0.052 μm were considered as *Cryptopsoridium parvum*. These findings were in accordance with the other workers [10].

**Figure-1:**
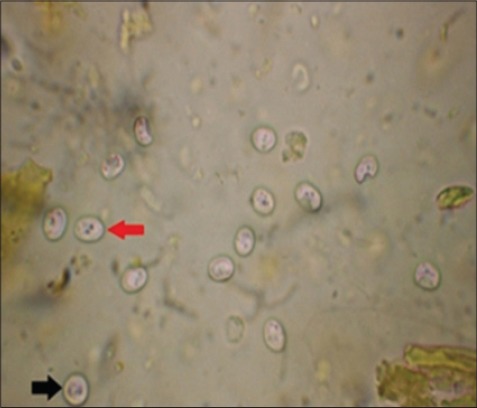
*Cryptosporidium parvum* (round red arrow), *Cryptosporidium andersoni* (oval black arrow) refractile oocyst under sheather's sugar floatation (×100).

In SSF, the oocyst appeared as round or oval, refractile bodies with a thin cytoplasmic membrane, finely granular cytoplasm (Figure-1). It was also described by Bowman [12]. Of all the techniques, fecal flotation with SSF was found to be a more sensitive method for the diagnosis of cryptosporidiosis which was in agreement with [12]. The sensitivity of the test is due to the concentration of oocysts in SSF. It gave significantly higher percentage of recovery even when the concentration of oocysts was low. However, when the oocysts were left in the hypertonic solution for more than 15 min, they begin to collapse and lose their spherical shape which was also quoted by Bowman [12]. Similar observations also made by Bhat *et al*. [18] when they used sugar flotation for detection of oocysts in Ludhiana.

**Figure-2:**
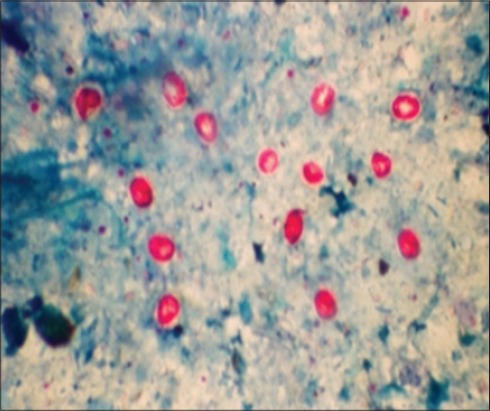
*Cryptosporidium parvum* (round) and *Cryptosporidium andersoni* (oval) pink oocysts in Ziehl–Neelsen staining.

In ZNs staining, the oocysts appeared as densely stained pink bodies against a pale green background, with a clear hallow around the oocysts with four whitish bar like naked sporozoites (Figure-2). The similar results were obtained with the hot and cold ZN staining. In India, this technique has been widely in use for diagnosis of cryptosporidiosis in animals [19-21]. Mucus portion of the fecal sample yielded more number of oocysts [1].

**Figure-3:**
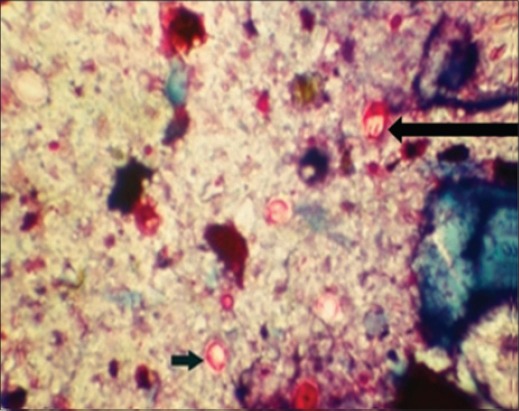
*Cryptosporidium parvum* (round) and *Cryptosporidium andersoni* (oval) red oocysts in Kinyoun's acid-fast staining method.

In Kinyoun's staining method, the oocysts appeared as red against the dark blue background and four sporozoites were visible (Figure-3). A common problem with ZN and Kinyoun's staining method is that they cannot differentiate *Cryptosporidium* oocysts from moulds and yeast [22]. Among the conventional staining methods both ZN (hot and cold) and Kinyoun's showed similar sensitivity. Similar observations were made by other workers [23] but both the methods were found to be time-consuming. Decolourization stage was found to be critical for both the techniques, which provided good contrast between oocysts and background. However, some oocysts did not get stained due to over exposure to decoloriser. In the hot method, shrinkage and distortion of oocysts were observed.

**Figure-4:**
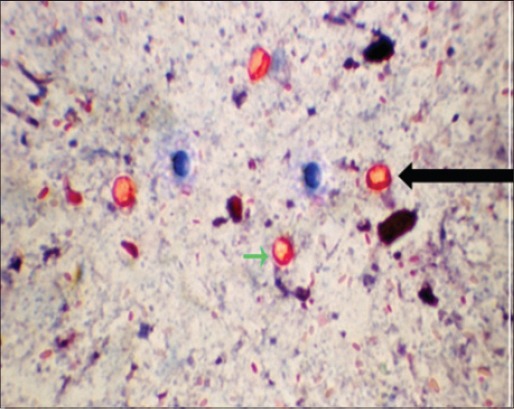
*Cryptosporidium parvum* (round) and *Cryptosporidium andersoni* (oval) oocysts in safranin methylene blue staining method.

In SMB oocysts appeared as orange-pink bodies and the sporozoites within the oocysts staind slightly darker. Yeast, bacteria, fungal spores and other fecal debris took the counter stain methylene blue in the SMB staining method. Thus, the method has got the advantage over other methods in differentiating oocysts from yeasts and moulds (Figure-4) [6].

**Figure-5:**
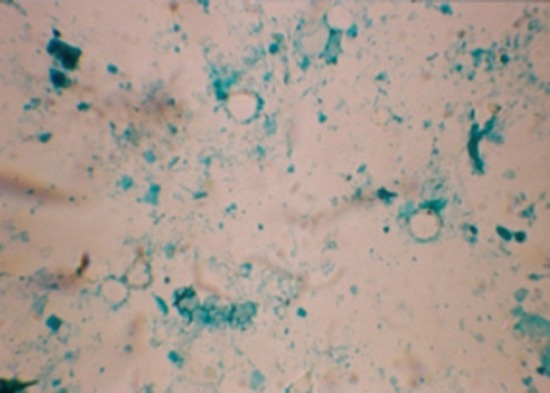
Negative staining of *Cryptosporidium oocyst* in light green (×100).

**Figure-6:**
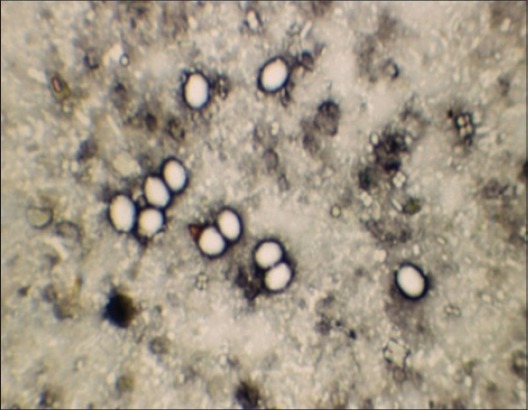
Negative staining of *Cryptosporidium oocyst* in nigrosin (×100).

In negative staining method, the oocysts were refractile and unstained against green background after light green staining (Figure-5), whereas in nigrosin staining, the oocysts were bright, refractile, unstained circular or oval bodies on a black background (Figure-6).

**Figure-7:**
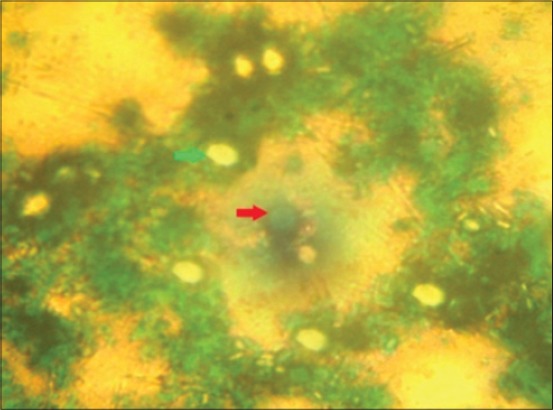
Appearance of *Cryptosporidium oocysts* and yeast in malachite green negative staining (×100). Green arrow: *Cryptosporidium oocysts*. Red arrow: Yeast

In malachite green-staining, oocysts appeared as plumpy and bright against dark green background (Figure-7). It was observed that in nigrosin and light green staining method, both oocyst and yeast remain unstained hence it was difficult for differentiation. But in the case of malachite green as yeast took up the stain they can be easily differentiated from oocysts which were in accordance with Chichino *et al*. [9]. Hence, in the present study, it was observed that negative staining with malachite green was superior over the other methods. Hence, it can be recommended for the rapid screening of a large number of samples which can be further confirmed by other techniques including molecular techniques to identify at the species level.

One-way ANOVA based on average oocyst counts in the SSF (8.0±0.528) and light green stained (1.65±0.297) smears showed a significant difference (p<0.05) when compared to all other methods. However, the difference between the mean oocyst counts among other techniques was non-significant. There was an increasing trend in mean oocyst count from nigrosin (3.327±0.359) to SMB staining method (5.072±0.487) as shown in Table-1.

**Table-1:** Average oocyst counts in different diagnostic methods (n=26 of 455 samples).

Staining methods	Average oocyst counts
SSF	8.000±0.528^c^
ZN (cold)	4.321±0.487^a^
ZN (hot)	4.250±0.498^a^
KAF	4.415±0.484^a^
SMB	5.072±0.459^a^
Malachite green	3.495±0.335^ab^
Light green	1.650±0.298^b^
Nigrosin	3.449±0.356^ab^

The mean values within a column bearing different superscript differ significantly (p≤0.05). ZN=Ziehl–Neelsen, SSF=Sheather’s sugar flotation, KAF=Kinyoun’s acid-fast method, SMB=Safranin-methylene blue staining

## Conclusions

In the present study, diagnosis of cryptosporidiosis in bovines caused by *C*. *parvum* and *C. andersoni* was carried out based on the detection and identification of oocysts in the fecal sample by employing various diagnostic techniques. Comparison of various diagnostic methods revealed that SSF was more sensitive and specific. The specific gravity of sucrose flotation fluid (1.26) and centrifugation at 500 ×*g* play important role in the recovery of oocysts. Among the conventional staining methods even though, there was no significant difference in the average oocyst counts, the SMB staining gives better differentiation of oocysts and yeast. In negative staining methods, malachite green staining technique gave a better results as it could differentiate between oocysts and yeasts. At present, among the available techniques, molecular techniques like PCR being the highly sensitive and specific test for the diagnosis of cryptosporidiosis. However, under field conditions where facilities are limited for carrying out PCR, concentration staining methods are recommended [23]. The study is helpful in conducting preliminary survey for screening of cryptosporidiosis in animals.

## Authors’ Contributions

The present study was a part of research work of HKMR during her M.V.Sc thesis program, Department of Parasitology. HKMR collected the samples, processed and thesis was written by her. The present paper is part of her research. GCP is her major advisor for guiding and thesis writing and PED is one of advisory member in the committee contributed in planning of research work and correction of thesis. GCP prepared the manuscript and PED corrected the draft and HKMR has finally reviewed the corrected copy for incorporation of all tests in the study. All authors read and approved the final manuscript.
